# Vaginal Transcriptional Signatures of the Neutrophil‐Driven Immune Response Correlate With Clinical Severity During Recurrent Vulvovaginal Candidiasis

**DOI:** 10.1111/aji.70040

**Published:** 2025-01-07

**Authors:** Tyra Hasselrot, Cathrin Alvendal, Sara Hunt, Mathias Franzén Boger, Vilde Kaldhusdal, Anastasios Damdimopoulos, Ina Schuppe‐Koistinen, Gabriella Edfeldt, Nina Bohm‐Starke, Kristina Broliden

**Affiliations:** ^1^ Department of Medicine Solna Division of Infectious Diseases Karolinska Institutet Department of Infectious Diseases Karolinska University Hospital Center for Molecular Medicine Stockholm Sweden; ^2^ Department of Clinical Sciences Division of Obstetrics and Gynecology Karolinska Institutet Danderyd Hospital Stockholm Sweden; ^3^ Bioinformatics and Expression Analysis Core Facility Karolinska Institutet Stockholm Sweden; ^4^ Department of Microbiology Tumor and Cell Biology (MTC) Centre for Translational Microbiome Research Karolinska Institutet Stockholm Sweden; ^5^ Science for Life Laboratory Karolinska Institutet Stockholm Sweden

**Keywords:** candida, epithelium, inflammation, transcriptomics, vagina

## Abstract

**Problem:**

Recurrent vulvovaginal candidiasis (RVVC) affects 5%−10% of all women, negatively impacting their reproductive health and quality of life. Herein, we investigated the molecular effects of RVVC on the vaginal mucosa of otherwise healthy women.

**Method of Study:**

Gene expression analysis was performed on vaginal tissue biopsies from women with RVVC, including those with a current episode of vulvovaginal candidiasis (VVC, *n* = 19) and women between infections (culture negative RVVC [CNR], *n* = 8); women asymptomatically colonized with *Candida albicans* (asymptomatic [AS], *n* = 7); and healthy controls (*n* = 18). Gene expression profiles were compared between groups and correlated with clinical data retrieved from questionnaires and gynecologic examinations.

**Results:**

Of 20 171 genes identified in vaginal biopsies, 6506 were differentially expressed in the RVVC group, compared to healthy controls. Gene expression pathway analysis revealed an association between RVVC and pathways of inflammatory responses, especially genes involved in neutrophil recruitment and activation. Expression of genes involved in inflammation and neutrophil recruitment increased with increasing clinical severity of VVC, whereas expression of some genes involved in epithelial integrity decreased with increasing clinical severity of infection. Gene expression profiles of both the CNR and AS groups were comparable to those of healthy controls.

**Conclusions:**

The clinical severity of RVVC during active infection correlates with increased expression of genes involved in molecular inflammation and neutrophil activation in the vaginal mucosa. The lack of differences between healthy controls and women with RVVC who were between acute infections indicates that the molecular effects observed in the RVVC group are only present during active infection.

## 1 Introduction

Vulvovaginal candidiasis (VVC) is an inflammatory condition of the vagina caused by infection with yeast, most commonly *Candida albicans*. VVC affects 70%−75% of women at least once during their lifetime [1, 2, 3]. The main symptoms are vaginal itching and soreness, abnormal vaginal discharge, and pain or discomfort during urination and/or sexual intercourse [2], all of which can have pronounced negative effects on quality of life, reproductive health, and sexual wellbeing. Approximately 5%−10% of women develop recurrent vulvovaginal candidiasis (RVVC), defined as persistent VVC or three or more episodes of VVC per year [1]. In addition to symptoms of acute VVC, patients with RVVC often present with additional diagnoses, such as depression, anxiety, or vulvodynia [4, 5].

Known risk factors for VVC include elevated estrogen levels, diabetes mellitus, immunosuppression, and use of antibiotics. However, the majority of women with RVVC lack any known triggers or risk factors for VVC and are thus diagnosed with idiopathic RVVC [2]. It has been hypothesized that women with idiopathic RVVC may have predisposing genetic alterations related to epithelial cell receptor function and inflammasome regulation, resulting in a hyperreactive inflammatory response that leads to VVC symptoms [6]. In VVC, an increased fungal burden, as well as the release of fungal toxins and proteases, triggers an innate immune response in vaginal epithelial cells through nuclear factor (NF)‐κB and mitogen‐activated protein kinase (MAPK) signaling pathways [6]. The subsequent production of proinflammatory cytokines and alarmins induces neutrophil recruitment and activation [7, 8]. The combination of high fungal burden and epithelial invasion, along with an aggressive innate immune response, is believed to result in the clinical symptoms of VVC, differentiating it from asymptomatic (AS) *C. albicans* colonization [9].

In our previous study, we investigated the transcriptional signatures of clinical VVC in the cervicovaginal mucosa of Kenyan sex workers and found that infection was associated with inflammation and neutrophil activation, but not with severe epithelial disruption [7]. Our present study aimed to further investigate the effects of culture‐verified VVC episodes on the vaginal mucosa of Swedish women with RVVC and to correlate these effects with the clinical severity of infection. Expanding our knowledge of the host molecular defense mechanisms in patients with RVVC is crucial to understanding the etiology of symptoms, as well as inadequate fungal clearance, in this condition.

## 2 Materials and Methods

### 2.1 Study Population

Women diagnosed with RVVC were recruited via the gynecology clinic at Danderyd's Hospital and primary care clinics in Stockholm, Sweden, as well as through flyers and advertising on social media platforms. Healthy women were also recruited through flyers and advertising on social media. All women were recruited between March 28, 2022 and December 8, 2023. The inclusion criteria for all study participants were age 18−46 years, not pregnant, no serious medical or gynecologic conditions (other than RVVC), and no antibiotics or antifungal agents during the previous 2 weeks.

At the enrollment visit, potential study participants completed a medical and social questionnaire. In the questionnaire, RVVC was defined as ≥3 episodes of self‐reported VVC during the past 12 months. All women also underwent a routine gynecologic examination by a senior gynecologist, which included obtaining urine for pregnancy testing and vaginal swabs for fungal cultures and polymerase chain reaction detection of chlamydia and gonorrhea. Two wet mounts of vaginal discharge (wet smears) were prepared with potassium hydroxide or saline to assess for signs of VVC (e.g., visible hyphae), vaginal dysbiosis (e.g., clue cells, reduced number of lactobacilli), and inflammation (e.g., leukocytosis), according to routine clinical procedures. Bacterial vaginosis was further evaluated using the Whiff test and pH test of vaginal swab samples, according to routine clinical procedures. Based on these evaluations, women diagnosed with bacterial vaginosis, chlamydia, gonorrhea, or infection with a fungal species other than *C. albicans* were excluded from the study.

Symptom scores and clinical scores were also assessed at the enrollment visit. Symptom scores (range, 0−5 points) were obtained by asking study participants whether they were currently experiencing vulvovaginal discharge (1 point), itching (1 point), dryness (1 point), burn (1 point), or pain (1 point). Clinical scores (range, 0−5 points) were based on the presence of vulvovaginal redness (1 point), discharge (1 point), dryness (1 point), or fissures (1 point) on gynecologic examination, as well as the presence of visible hyphae in wet mount (1 point). Women with RVVC were divided into two groups: women with a current VVC episode (positive culture for *C. albicans* and symptom score ≥1) and women with a history of RVVC but negative fungal cultures. Similarly, healthy women were divided into two groups: women with no symptoms and negative fungal cultures and women asymptomatically colonized with *C. albicans* (fungal culture positive for *C. albicans* and symptom score of 0).

### 2.2 Fungal Cultures

Vaginal swabs were transported in liquid amies transport medium, cultured on CHROMagar Candida Plus and incubated at 37°C for 24–48 h. *C. albicans* typically appears as green colonies. To confirm species identification, MALDI‐TOF was done. Antifungal susceptibility testing was performed using YeastOne Sensititre (Thermo Fisher Scientific, Waltham, MA, USA), according to the manufacturer's recommendation. When MIC‐values were classified as “Susceptible, increased exposure” (I) or “Resistant” (R), verification was performed using the EUCAST broth microdilution method [10].

### 2.3 Collection of Vaginal Biopsies

Following the collection of vaginal swab samples for diagnosing sexually transmitted infections (STIs), bacterial vaginosis, and VVC, vaginal biopsies were obtained. An experienced gynecologist used Schubert biopsy forceps to collect two 3‐mm biopsy specimens from the right vaginal wall, approximately 3 cm from the introitus. The specimens were placed in FluidX tubes (Brooks Life Sciences, Chelmsford, MA, USA) containing 0.8 mL DNA/RNA‐shield (Zymo Research, Irvine, CA, USA) [11], snap frozen in ice‐cold ethanol within 1 min, and subsequently stored at −80°C within 10 min.

### 2.4 Bulk RNA‐Sequencing

After the vaginal specimens preserved in DNA/RNA‐shield were thawed, total RNA was isolated. The RNA was subsequently purified using the AllPrep DNA/RNA Mini Kit (QIAGEN, Hilden, Germany) and QIAcube Connect (QIAGEN). RNA quantities were determined using a NanoDrop ND‐1000 spectrophotometer (Thermo Fisher Scientific, Waltham, MA, USA), and the RNA integrity number (RIN) was assessed using the Agilent 2200 TapeStation System (Agilent Technologies, Santa Clara, CA, USA). Samples with RIN <7.0 were excluded from the analysis. TruSeq mRNA‐Seq library prep kit (Illumina, San Diego, CA, USA) was used for mRNA purification and subsequent conversion into cDNA libraries (using reverse transcriptase, random primers, and DNA polymerase I). Barcoded cDNA libraries were pooled and loaded onto reagent cartridges (Illumina) and sequenced in a NextSeq 550 (Illumina).

### 2.5 Differential Gene Expression, Gene Set Enrichment Analysis (GSEA), and Clinical Score Analysis

Genes differentially expressed between study groups were assessed using a generalized linear model (glm) from the EdgeR package (19910308) [12]. Differentially expressed genes (DEGs) with a false‐discovery rate (FDR)—adjusted *p* value <0.05 were considered statistically significant. GSEA was performed on all DEGs to predict functional pathways. Upregulated and downregulated genes were analyzed both separately and in combination. The DEGs underwent GSEA against the databases gene ontology (GO) [13, 14], kyoto encyclopedia of genes and genomes (KEGG) [15, 16], WikiPathways [17], Reactome [18], and the molecular signatures database (MSigDB) [19]. Furthermore, to assess clustering of samples based on gene expression, hierarchical clustering analysis and uniform manifold approximation and projection (UMAP) analysis were performed based on all DEGs. Clinical data were compared between UMAP clusters using the Mann–Whitney *U*‐test for continuous variables, and Fisher's exact test for binary variables.

To assess correlations between gene expression and clinical severity of RVVC, the clinical score was used as a continuous variable, and the glm model was used to identify genes with expression rates following the same trend as the clinical score of the study participants. Genes with an FDR‐adjusted *p* value <0.05 were considered statistically significant. GSEA was performed on all genes following the clinical score trend against the databases GO, KEGG, WikiPathways, Reactome, and MSigDB, as described above for all DEGs.

## 3 Results

### 3.1 Clinical Characteristics of Study Participants

**FIGURE 1:**
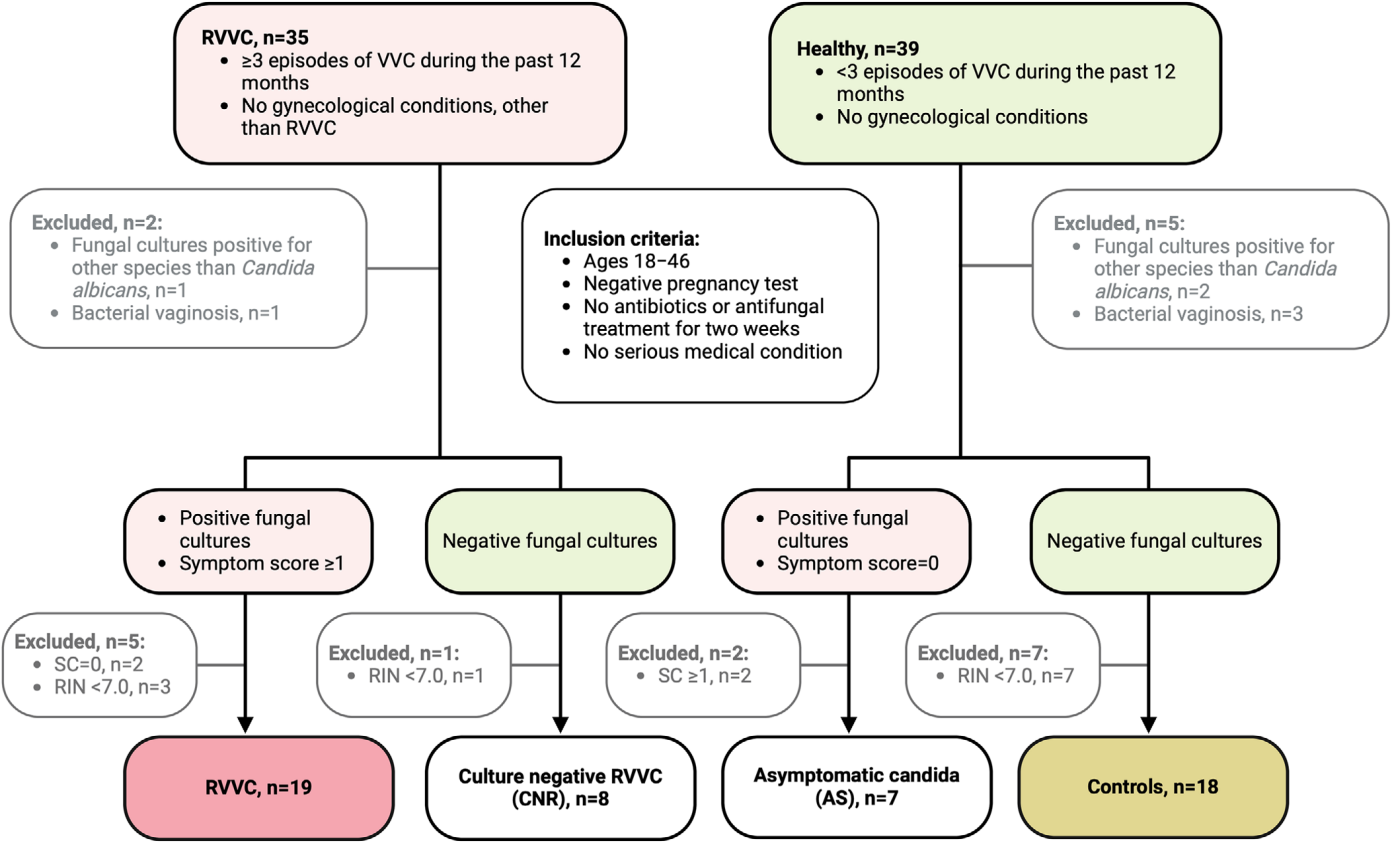
Inclusion criteria and stratification of study groups. Symptom score (0−5): vulvovaginal discharge = 1p, itching = 1p, dryness = 1p, burn = 1p, pain = 1p, self‐reported. RIN indicates RNA integrity number; RVVC, recurrent vulvovaginal candidiasis; SC, symptom score; VVC, vulvovaginal candidiasis.

A total of 74 women were recruited for this study and attended the enrollment visit. Eighteen women were subsequently excluded because of vaginal cultures positive for fungal species other than *C. albicans* (*n* = 3), bacterial vaginosis (*n* = 4), or low quality (RIN <7.0) of RNA isolated from biopsy samples (*n* = 11) (Figure 1). Women with a fungal culture positive for *C. albicans*, symptom score ≥1, and history of ≥3 episodes of self‐reported VVC during the past 12 months were defined as the RVVC group (*n* = 19). Women with a negative fungal culture and no history of RVVC were defined as the healthy control group (CTRL group, *n* = 18). Women initially recruited as healthy controls (i.e., no history of RVVC) who had a current symptom score of 0 but a fungal culture positive for *C. albicans* were defined as the asymptomatically colonized study group (AS group, *n* = 7). Women initially recruited for the RVVC group (history of ≥3 episodes of self‐reported VVC during the past 12 months) but whose current fungal culture was negative were defined as the culture‐negative RVVC study group (culture negative RVVC [CNR] group, *n* = 8) (Figure 1).

The median age of study participants was 30 years (range, 20−46 years), and the age did not differ significantly between study groups (Table 1). Body mass index based on self‐reported height and weight also did not differ significantly between groups. All women were generally healthy, except for two women in the CTRL group: one was diagnosed with asthma and the other with depression. Regular menstruations were more commonly reported in the AS group as compared to the CTRL group (86% and 33%, respectively, *p* = 0.03), and use of hormonal intrauterine device (IUD) as method of contraception was less common in the CNR group as compared to the CTRL group (0% and 50%, respectively, *p* = 0.03). Based on self‐reported symptoms, the median symptom score was four in the RVVC group (range, 2−5 [women with symptom score = 0 were not included in the RVVC group]) and 0 (range, 0−1) in the CTRL group. The median gynecologist‐assessed clinical score was three in the RVVC group (range, 0−5), whereas the clinical score was 0 for all women in the CTRL group (Table S1).

**TABLE 1:** Clinical data based on self‐report through questionnaires. Women with positive fungal cultures and ≥3 episodes of VVC per year were defined as an RVVC group, while women with negative fungal cultures and <3 episodes of VVC per years were defined as controls. Women with positive fungal cultures, symptom score = 0, and <3 episodes of VVC per year were defined as an asymptomatic group. Women with negative fungal cultures and ≥3 episodes of VVC per year were defined as a culture negative RVVC group.

	Median or number (range or %)				*p* value^a^				Data n/a			
	RVVC *n* = 19	CTRL *n* = 18	AS *n* = 7	CNR *n* = 8	RVVC/Cntrl	AS/Cntrl	CNR/Cntrl	RVVC/CNR	RVVC Ctrls AS CNR			
Age (years)	32 (21−46)	32 (23−42)	27 (20−37)	29 (26−45)	0.75	0.32	0.82	>0.9				1
BMI	22 (19−27)	24 (16−36)	22 (18−23)	22 (20−24)	0.28	0.24	0.42	>0.9	1	1		2
Smoker	0 (0)	2 (11)	1 (14)	0 (0)	0.23	>0.9	>0.9	>0.9				2
Healthy	19 (100)	16 (89)	7 (100)	7 (100)	0.23	>0.9	>0.9	>0.9				1
Married/stable relationship	15 (79)	14 (78)	4 (57)	5 (83)	>0.9	0.36	>0.9	>0.9				2
No. of pregnancies	2 (0−5)	1 (0−5)	0 (0−1)	1 (0−2)	0.64	0.05	0.51	0.25	2	2	1	2
No. of children	1 (0−3)	1 (0−3)	0 (0−1)	0 (0−1)	0.53	0.16	0.37	0.16	2	2	1	2
Regular menstruations	13 (68)	6 (33)	6 (86)	3 (50)	0.05	0.03*	0.63	0.63				2
**Contraceptive use**												
Hormonal IUD	6 (32)	9 (50)	1 (14)	0 (0)	0.32	0.18	0.03*	0.15				1
Birth controll pills	6 (32)	3 (17)	3 (43)	3 (43)	0.45	0.30	0.30	0.66				1
No contraceptive	7 (37)	5 (28)	3 (43)	4 (57)	0.73	0.64	0.20	0.41				1
**History of sexually transmitted infections**												
Chlamydia	6 (32)	4 (22)	1 (14)	3 (50)	0.71	>0.9	0.31	0.63				2
Herpes	1 (5)	0 (0)	0 (0)	1 (17)	>0.9	>0.9	0.25	0.43				2
Condyloma	4 (21)	0 (0)	0 (0)	0 (0)	0.11	>0.9	>0.9	0.54				2
Foul‐smelling vaginal discharge	5 (26)	4 (22)	0 (0)	2 (33)	>0.9	0.30	0.62	>0.9				2
Cervical dysplasia	6 (32)	3 (17)	1 (14)	2 (33)	0.45	>0.9	0.57	>0.9				2
**RVVC**												
Duration of RVVC (years)	5 (0.4−20)	na	na	5 (4−13)	na	na	na	0.67	1			4

Abbreviations: AS, asymptomatic; BMI, body mass index (kg/m^2^); CNR, culture negative RVVC; CTRL, controls; IUD, intrauterine device; n/a, not available (excluded from statistical analysis); no, number; RVVC, recurrent vulvovaginal candidiasis.

^a^ A Mann–Whitney *U* test was applied for continous variables, and Fischer's exact test was applied for binary variables. *p* < 0.05 was considered statisticaly significant, significant *p* values are marked with an asterisk.

### 3.2 RVVC is Associated With Gene Expression Profiles Consistent With Neutrophil Recruitment and Immune Activation

To investigate gene expression associated with RVVC, we used RNA‐sequencing analysis to define the host transcriptomic profile of 52 vaginal tissue samples. A total of 20 171 genes were detected in the dataset. Genes with an FDR‐adjusted *p* value <0.05 were considered significantly differentially expressed between study groups. The RNA expression profiles were first stratified for the two main study groups (RVVC, *n* = 19; CTRL, *n* = 18). The transcriptional analysis revealed 6506 DEGs between the RVVC and CTRL groups. Among these DEGs, 3649 were upregulated (log_2_ fold change [FC] range, 0.12–9.4) and 2857 were downregulated (log_2_FC range, −0.12 to −4.8) in the RVVC group, compared to the CTRL group (Tables 2 and S2).

**TABLE 2:** Number of differentially expressed genes identified when comparing the study groups, and number of genes following the same trend as the clinical score. Genes were identified using the glm pipeline from the EdgeR package.

	Upregulated genes	Downregulated genes	Non‐significant genes
RVVC (*n* = 19) versus CTRL (*n* = 18)	3649	2857	13 665
AS (*n* = 7) versus CTRL (*n* = 18)	3	0	20 168
CNR (*n* = 8) versus CTRL (*n* = 18)	0	0	20 171
RVVC (*n* = 19) versus CNR (*n* = 8)	947	602	18 622
Genes following the same trend as the clinical score	4421	4565	10 523

Genes with an FDR‐adjusted *p* value <0.05 were considered statistically significant genes.

Abbreviations: AS, asymptomatic; CNR, culture negative RVVC; FDR, false discovery rate; GLM, generalized linear model; RVVC, recurrent vulvovaginal candidiasis.

The upregulated DEGs in the RVVC group included genes involved in immune activation and neutrophil recruitment, such as genes encoding pathogen recognition receptors (e.g., *CLEC*s, *TLR*s, *NLR*s, *MCR1*), proinflammatory mediators (e.g., *IL1B, IL17A, IFNG, NOS2*, *CSF3*), neutrophil‐attracting chemokines (e.g., *CXCL1−3, CXCL6, CLXCL8−11, CXCL13, CXCL16, CCL2, CCL7−8*), and the neutrophil receptor *CXCR1*, as well as antimicrobial peptides and enzymes (e.g., *LTF, DEFB4A, DEFB4B, LYZ*, *S100A7A*) (Table S2). The downregulated genes in the RVVC group included genes involved in epithelial integrity (*CDSN*, *KPRP*, *KRT1*, and *FLG*), as well as several genes encoding cytochrome P450 enzymes (*CYP2A6‐7*, *CYP2C9*, *CYP2C18*, *CYP2C19*, *CYP2J2*, *CYP2W1*, *CYP3A4*, *CYP4F12*, *CYP4F22*, *CYP46A1*) (Table S2). The 15 most upregulated and downregulated DEGs according to log_2_FC values are presented in Table 3.

**TABLE 3:** The most differentially expressed genes between the RVVC (*n* = 19) and CTRL (*n* = 18) group, according to log_2_FC. Genes without HGNC ID are presented with ensembl ID.

HGNC ID	Gene name	Uniprot ID	Log_2_FC	FDR‐adj. *p* value
**Top 15 most upregulated genes in the RVVC group**				
CXCL1	C‐X‐C motif chemokine ligand 1	P09341	9.4	4.7x‐10^12^
CXCL8	C‐X‐C motif chemokine ligand 8	P10145	9.0	2.2x‐10^10^
IL19	Interleukin 19	Q9UHD0	8.5	2.5x‐10^8^
IL22	Interleukin 22	Q9GZX6	7.9	1.3x‐10^6^
LTF	Lactotransferrin	P02788	7.7	1.4x‐10^10^
CXCL6	C‐X‐C motif chemokine ligand 6	P80162	7.6	3.7x‐10^9^
NOS2	Nitric oxide synthase 2	P35228	7.3	5.5x‐10^8^
CSF3	Colony stimulating factor 3	P09919	6.6	1.0x‐10^7^
S100A7A	s100 calcium binding protein A7A	Q86SG5	6.5	7.7x‐10^7^
MMP7	Matrix metallopeptidase 7	P09237	6.5	2.7x‐10^7^
TRIM40	Tripartite motif containing 40	Q6P9F5	6.3	6.1x‐10^5^
CXCL13	C‐X‐C motif chemokine ligand 13	O43927	6.2	1.2x‐10^5^
OSM	Oncostatin M	P13725	6.2	3.8x‐10^10^
LILRA5	Leukocyte immunoglobulin like receptor A5	A6NI73	5.9	2.3x‐10^8^
CLEC4D	C‐type lectin domaine family 4 member D	Q8WXI8	5.8	1.2x‐10^6^
**Top 15 most downregulated genes in the RVVC group**				
CDSN	Corneodesmosin	Q15518	−4.8	7.3x‐10^7^
Ensembl ID: ENSG00000263427		—	−3.2	5.3x‐10^3^
Ensembl ID: ENSG00000258837		—	−3.1	1.7x‐10^3^
CYP2F2P	Cytochrome P450 family 2 subfamily F member 2, pseudogene	—	−3.0	5.0x‐10^3^
KRT36	Keratin 36	O76013	−2.8	9.3x‐10^5^
HTR2C	5‐Hydroxytryptamine receptor 2C	P28335	−2.8	6.4x‐10^3^
CYP2A6	Cytochrome P450 family 2 subfamily A member 6	P11509	−2.7	5.3x‐10^3^
C5orf46	Chromosome 5 open reading frame 46	Q6UWT4	−2.6	1.6x‐10^3^
CHGA	Chromogranin A	P10645	−2.5	9.8x‐10^3^
SERPINA12	Serpin family A member 12	Q8IW75	−2.5	3.0x‐10^3^
TMEM72	Transmembrane 72	A0PK05	−2.4	5.5x‐10^4^
GDNF‐AS1	GDNF antisense RNA 1	—	−2.4	8.8x‐10^5^
ELFN2	Extracellular leucine rich repeat and fibronectin type III domain containing 2	Q5R3F8	−2.4	6.7x‐10^7^
CYP2A7	Cytochrome P450 family 2 subfamily A member 7	P20853	−2.3	2.5x‐10^2^
SULT1C3	Sulfotransferase family 1C member 3	Q6IMI6	−2.3	5.2x‐10^3^

Abbreviations: CTRL, controls; FDR adj., false discovery rate adjusted; HGNC, HUGO gene nomenclature committee; log_2_FC, log_2_(fold change); RVVC, recurrent vulvovaginal candidiasis.

### 3.3 RVVC is Associated With Functional Pathways of Immune Activation and Response to Infection

**FIGURE 2:**
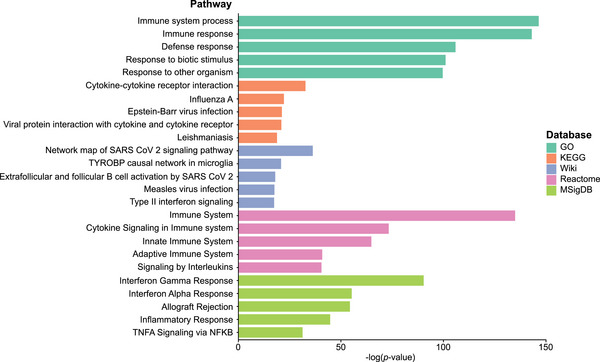
Gene set enrichment analysis of all differentially expressed genes. Top five pathways most significantly associated with the 3649 upregulated genes (FDR‐adjusted *p* value <0.05, log_2_FC >0) between the RVVC (*n* = 19) and CTRL (*n* = 18) group when compared against the databases GO, KEGG, WikiPathways, Reactome, and MsigDB. CTRL indicates controls; GO, gene ontology; KEGG, Kyoto encyclopedia of genes and genomes; MSigDB, the molecular signatures database; RVVC, recurrent vulvovaginal candidiasis; Wiki, WikiPathways.

GSEA was performed on all 6506 DEGs identified between the RVVC and CTRL groups, using the GO, KEGG, WikiPathways, Reactome, and MSigDB databases to identify functional pathways associated with the gene expression profile of the RVVC group. Upregulated and downregulated genes were analyzed both separately and in combination (Table S3). The most significant pathways associated with upregulated genes in the RVVC group (compared to the CTRL group) were immune response pathways, inflammatory pathways, and pathways involved in responses to infectious diseases. The downregulated genes were mainly associated with metabolic pathways. The five most significant pathways according to *p* values from each database were all associated with upregulated genes and are shown in Figure 2.

### 3.4 Clustering Analysis Based on DEGs

**FIGURE 3:**
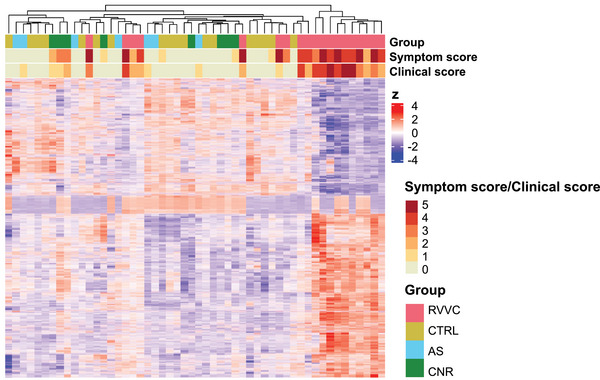
Hierarchial clustering of differentially expressed genes. Heatmap of all 6506 DEGs (FDR‐adjusted *p* value <0.05) between the RVVC (*n* = 19) and CTRL (*n* = 18) group. Each study participant is represented by a vertical column and each gene is represented by a horizontal row. The expression of each gene is standardized (*z*) to a mean of 0 and a standard deviation of 1. Red: above average expression, blue: below average expression. AS indicates asymptomatic; CNR, culture negative RVVC; CTRL, controls; DEGs, differentially expressed genes; FDR, false‐discovery rate; RVVC, recurrent vulvovaginal candidiasis.

**FIGURE 4:**
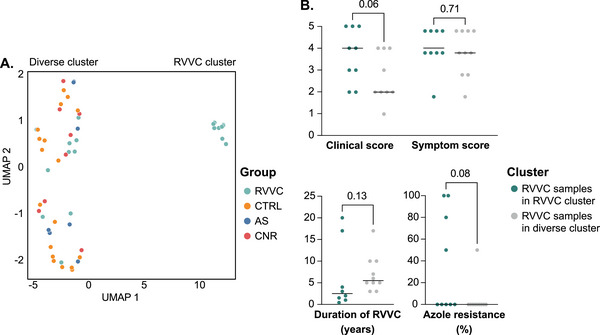
UMAP of all samples based on gene expression. UMAP based on all 6506 DEGs (FDR‐adjusted *p* value <0.05) between the RVVC (*n* = 19) and CTRL (*n* = 18) group (A). Statistical comparison of clinical score (assessed by gynecologist), symptom score (self‐reported), duration of RVVC (self‐reported) and azole resitance (assessed according to clinical routine, and measured as the percentage of tested azoles the yeast proved resistant against) between RVVC samples in the RVVC cluster and RVVC samples in the diverse cluster (B). AS indicates asymptomatic; CNR, culture negative RVVC; CTRL, controls; DEGs, differentially expressed genes; FDR, false‐discovery rate; RVVC, recurrent vulvovaginal candidiasis; UMAP, uniform manifold approximation and projection.

Hierarchical clustering analysis based on differential gene expression between the RVVC and CTRL groups revealed one group containing samples from women in the RVVC group with varying symptom and clinical scores and one group consisting of samples from all four study groups (Figure 3). UMAP analysis was performed based on all DEGs between the RVVC and CTRL groups to further assess clustering of samples. This analysis revealed two distinct clusters: one cluster consisted of nine samples from the RVVC group, and the other cluster contained the remaining 10 RVVC samples interspersed with all samples from the CTRL, AS, and CNR groups (Figure 4A).

To investigate differences between RVVC samples in the RVVC cluster and RVVC samples clustering with the CTRL group, statistical analyses were performed to compare clinical features between these two groups based on data retrieved from the enrollment questionnaires and gynecologic examinations (Table S4). These analyses revealed that the samples in the RVVC cluster were obtained from women with higher clinical scores than the women whose samples clustered with the CTRL group, although the difference was not statistically significant (*p* = 0.06). Symptom scores were similar between RVVC samples in the two clusters. Azole resistance was trending toward higher in the unique RVVC cluster (*p* = 0.08), while duration of RVVC was similar between clusters (Figure 4B, Table S4).

### 3.5 Dynamic Expression of Inflammatory Genes Correlates With Clinical Severity of VVC

**FIGURE 5:**
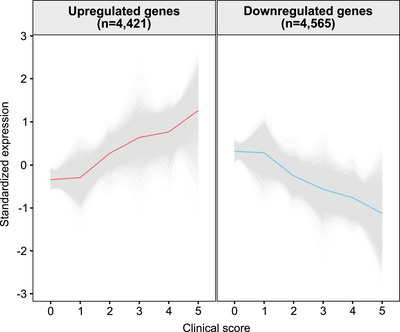
Genes significantly associated with clinical severity of RVVC. The clinical score was used as a continuous variable to identify up‐ and downregulated genes, respectively, with expression rates following the same trend as the clinical score. Genes with an FDR‐adjusted *p* value <0.05 were considered statistically significant. All genes are in grey, and the average expression is shown in red/blue. FDR indicates false‐discovery rate; RVVC, recurrent vulvovaginal candidiasis.

**FIGURE 6:**
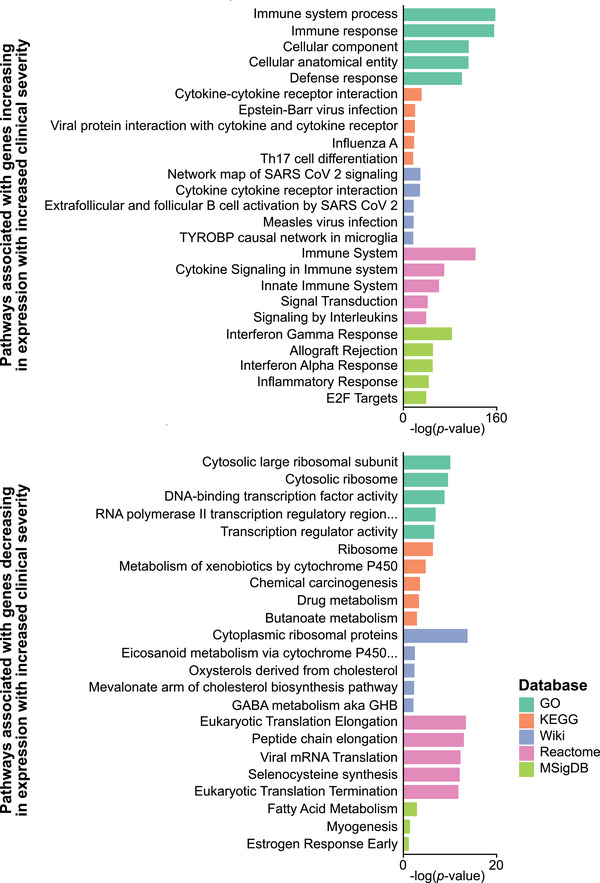
Gene set enrichment analysis of genes associated with clinical severity of RVVC. Top five pathways most significantly associated with the genes following the clinical score trend, when compared against the databases GO, KEGG, WikiPathways, Reactome, and MsigDB. GO indicates gene ontology; KEGG, kyoto encyclopedia of genes and genomes; MSigDB, the molecular signatures database; Wiki, WikiPathways.

As a trend toward higher clinical scores was observed in the RVVC cluster, the clinical score of the study participants was used as a continuous variable to identify genes with expression rates following the same trend as the clinical score. In this way, the relationship between gene expression and clinical severity of RVVC could be assessed. The analysis revealed 4421 upregulated genes with expression rates positively correlating with clinical score (log_2_FC range, 0.02–2.29), and 4565 downregulated genes negatively correlating with clinical score (log_2_FC range, −0.02 to −1.62) (Tables 2 and S5, Figure 5). The upregulated genes included genes encoding neutrophil‐attracting chemokines (*CXCL1‐3, 6, 8‐11, 13*, and *16*), the neutrophil receptor *CXCR1*, proinflammatory mediators (*IL1B*, *IL17A*, *IFNG*, *NOS2*, and *CSF3*), and antimicrobial peptides (*LTF*, *LYZ*, and *S100A7*). The downregulated genes included epithelial barrier marker genes (*CDSN*, *KPRP*, *KRT1*, and *FLG*) (Table S5). GSEA of the genes following the clinical score trend revealed the upregulated genes to be associated with pathways of immune system processes, while the downregulated genes were associated with mainly metabolic and structural pathways (Figure 6, Table S6). Collectively, these results highlight the dynamic nature of proinflammatory gene expression, which correlated with the degree of inflammation visualized clinically.

### 3.6 The Vaginal Transcriptome in Women With Asymptomatic Colonization is Comparable to That of Healthy Controls

To investigate the effects of asymptomatic colonization with *C. albicans* on the vaginal mucosa, the group of women asymptomatically colonized with *C. albicans* (AS group, *n* = 7) was compared to the CTRL group (*n* = 18). Transcriptional analysis revealed only three DEGs (*MTCO3P12*, *IGHV3‐74*, *MTCO1P12*) between the AS and CTRL groups, all of which were upregulated in the AS group (log_2_FC range, 1.7–4.8) (Tables 2 and S2). In the hierarchical clustering and UMAP analyses, the AS group clustered with the CTRL group (Figures 3 and 4A).

### 3.7 The Vaginal Transcriptome Between VVC Episodes is Comparable to That of Healthy Controls

To investigate the state of the vaginal mucosa between episodes of infection in women with RVVC, the group of women with a history consistent with RVVC but whose current fungal culture was negative (CNR, *n* = 8) was compared to the CTRL group. Transcriptional analysis revealed no DEGs between the CNR and CTRL groups (Tables 2 and S2), and in the hierarchical clustering and UMAP analyses, the CNR group clustered with the CTRL group (Figures 3 and 4A). When comparing the RVVC group (*n* = 19) to the CNR group, analysis revealed 1549 DEGs between the groups. Among these DEGs, 947 were upregulated (log_2_ FC range, 0.2–7.2) and 602 were downregulated (log_2_FC range, −0.2 to −4.2) in the RVVC group, compared to the CNR group. Out of these 1549 genes, 1417 genes were also found among the DEGs between the RVVC and CTRL groups (Tables 2 and S2).

## 4 Discussion

In this study, we investigated the effects of *C. albicans* cultur—positive RVVC on the vaginal mucosa of otherwise healthy women. Analysis of vaginal tissue biopsies from women currently experiencing an episode of RVVC showed upregulation of genes involved in neutrophil recruitment and immune activation, as assessed through transcriptomic profiling.

Among the upregulated genes associated with RVVC, we found several genes encoding C‐type lectin receptors (CLRs), the most predominant fungal‐sensing pathogen recognition receptors in humans [20]. The most upregulated CLR gene was *CLEC4D*, which encodes C‐type lectin/dectin‐3, a receptor that recognizes a‐mannans on the surface of *C. albicans* hyphae [21]. CLR binding stimulates an inflammatory response, including activation of the NF‐kB pathway and formation of the NOD‐like receptor protein 3 (NLRP3) inflammasome, leading to the release of proinflammatory cytokines and chemokines [20]. In VVC, the NLRP3 inflammasome is important for neutrophil recruitment and cytokine production [22, 23, 24]. In the current study, we observed upregulation of the genes for several NLRs, including the *NLRP3* gene. Activation of the NLRP3 inflammasome in VVC is inhibited by the interleukin (IL)‐22/NLRC4/IL‐1 receptor antagonist (IL‐1Ra) axis, and reduced levels of IL‐1Ra and IL‐22 have been observed in vaginal fluid from women with RVVC [24]. Interestingly, we found upregulation of both *IL22* and *NLRC4* in vaginal tissue samples from women with RVVC but no difference in *IL1RN* expression (the gene encoding IL‐1Ra) between women with RVVC and healthy controls. These discrepancies may reflect differences between gene expression in tissues and cytokine secretion in vaginal fluids. Activation of inflammasomes leads to proteolytic cleavage of IL‐1b and gasdermin D [25], the genes for which (*IL1B* and *GSDMD*) were both upregulated in our RVVC group. Subsequently, IL‐1b, together with other proinflammatory mediators (IL‐17, interferon‐gamma, and nitric oxide synthase 2), may induce the secretion of antimicrobial peptides, such as lactoferrin and S100‐A7A [26]. The genes for all of these proinflammatory mediators and peptides were upregulated in the RVVC group. The protective role of IL‐17 in oral candida infections is well established [27, 28], while the role of IL‐17 in VVC remains more controversial. In mice models, IL‐17 has been reported to play a protective role in RVVC through neutrophil regulation in some studies [29, 30], while others have shown no effect of IL‐17 on fungal burden or vaginal neutrophil infiltration [31, 32]. In our study, we found *IL17A* (the gene encoding IL‐17) to be among the most upregulated genes in women suffering from RVVC as compared to controls, indicating that IL‐17 plays an important role in human VVC.

Several genes encoding neutrophil‐attracting chemokines in the CXC‐ligand family were upregulated in the RVVC group. Among these was the gene encoding C‐X‐C motif chemokine ligand 8 (*CXCL8*), which is the most potent human neutrophil‐attracting chemokine. It is produced by various cells in response to infectious or inflammatory stimuli [26, 33]. Additionally, we observed upregulation of C–C motif chemokine ligand 2 (*CCL2*), *CCL7*, and *CCL8*. The proteins encoded by these genes are all weak neutrophil attractants by themselves but have been shown in vitro to synergize with CXCL8 to effectively stimulate neutrophils [34]. CXC‐ligands activate neutrophils through the CXCR1 and CXCR2 receptors, leading to chemotaxis, degranulation, and autocrine CXC‐ligand secretion [33, 35]. The upregulated genes in our RVVC group included *CXCR1*, as well as genes encoding antimicrobial peptides within neutrophil granules, such as lactoferrin (*LTF* gene), human β‐defensin‐4 (*DEFB4* gene), and lysozyme (*LYZ* gene), all of which have antifungal activity against *C. albicans* [36, 37, 38, 39]. Furthermore, one of the most upregulated genes in our study participants with RVVC was *CSF3*, which encodes colony‐stimulating factor 3, a cytokine essential for neutrophil production and function [40]. Interestingly, it has previously been reported that neutrophils from women with RVVC have reduced fungicidal activity and proliferative capacity, compared to healthy controls [41], which may explain why activation of a neutrophil‐driven immune response does not lead to fungal clearance in patients with RVVC. The mechanism of neutrophil dysfunction has, in mice, been shown to be due to inhibiting factors present in the vaginal microenvironment and has further been linked to increased vaginal tissue damage [42]. Upregulation of genes involved in inflammatory responses and immune signaling was broadly reflected in the pathways found in the GSEA. The most significant pathways found in all five databases were related to immune system processes.

Cluster analysis of vaginal mucosal samples based on gene expression revealed that approximately half of samples from women in the RVVC group clustered separately from the other samples (including samples from the healthy controls and the remaining RVVC group women). When comparing clinical data between these two clusters, we found that, in addition to the trend of higher clinical scores in the unique RVVC cluster, the incidence of azole resistance was trending toward higher in this RVVC cluster. Higher azole resistance could be due to prolonged or inefficient use of antifungal agents; however, the duration of RVVC (i.e., self‐reported years since the onset of fungal infections) did not differ between cluster groups. Alternatively, these women may have been infected with resistant strains of *C. albicans*. In a recent study by Gerges et al. (2023), azole resistance was associated with increased biofilm formation and proteinase production (both of which are important virulence factors) in clinical *C. albicans* isolates from women with VVC in Egypt [43]. Conversely, a similar study on *C. albicans* isolates from women with VVC in India found no significant relationship between azole resistance and biofilm formation or proteinase production, and other studies on vaginal and nonvaginal *C. albicans* isolates have even shown an association between antifungal resistance and decreased virulence [44, 45, 46]. In the current study, azole resistance was measured as the number of azoles the yeast was resistant to during standard clinical resistance determination, and more comprehensive evaluation of azole resistance should be conducted in future studies to fully understand its role in RVVC.

When assessing gene expression in relation to clinical severity of RVVC, we found that expression of several genes increased as RVVC severity increased, including *CXCL1−3*, *6*, *8*, *13*, and *16*, as well as *CXCR1* and genes encoding several proinflammatory mediators and antimicrobial peptides. These results suggest that recruitment and activation of neutrophils is a dynamic process, and that the severity of inflammation observed clinically in RVVC correlates with levels of proinflammatory gene expression. Furthermore, expression of *CDSN*, *KPRP*, *KRT1*, and *FLG* decreased with increasing clinical severity. These genes encode proteins demonstrated to have important roles in maintaining epithelial integrity at other mucosal sites [47, 48, 49, 50, 51], suggesting that the clinical severity of RVVC may correlate with a less robust epithelial barrier. Decreased epithelial integrity caused by genital inflammation is associated with an increased risk of acquiring STIs, including human immunodeficiency virus (HIV) in endemic regions [52]. The neutrophil‐driven inflammation associated with bacterial vaginosis is minimally visible to clinicians (unlike that of RVVC) but has been linked to downregulation of epithelial junction proteins and an increased risk of acquiring HIV [53, 54, 55]. Epithelial disruption in RVVC may be further mediated by fungal release of proteolytic enzymes and toxins [56, 57, 58, 59], as well as through epithelial penetration of *C. albicans* hyphae [9, 60–62]. These observations highlight the importance of ensuring proper RVVC treatment and adequate fungal clearance in women at risk of acquiring STIs.

We also assessed vaginal tissue biopsies from women with RVVC who were not currently experiencing an episode of VVC (i.e., the CNR group). Transcriptomic profiling revealed that the vaginal mucosa of these women was comparable to that of healthy controls. Moreover, over 90% of the DEGs identified between the CNR and RVVC group were also found among the DEGs between the RVVC group and healthy controls. This suggests that the mucosa may recover between infections and that the molecular effects observed in our RVVC group are only present during active VVC episodes. The lack of difference between the CNR and control groups in this study indicates the need to look beyond gene expression to identify possible predisposing factors for idiopathic RVVC.

*C. albicans* is an opportunistic pathogen, and it is estimated that 10%−20% of women are asymptomatically colonized with the organism in the cervicovaginal mucosa [1]. Current knowledge is limited as to why *C. albicans* might asymptomatically colonize some women and cause symptomatic infection in others. Some studies suggest that the difference in symptomatology is primarily dependent on inherent fungal abilities, such as filamentation, immune evasion, or biofilm formation [62, 63]. Other research suggests that susceptibility to VVC depends more on host factors, such as a dysregulated immune response, impaired epithelial barrier function, or an imbalanced vaginal microbiome [8, 64–66]. Interestingly, we found that gene expression in the vaginal mucosa was similar between women asymptomatically colonized with *C. albicans* and healthy controls, whereas approximately one‐third of all detected genes were differentially expressed between women with symptomatic VVC and controls. Although these findings do not explain why *C. albicans* causes asymptomatic colonization in some women and symptomatic infection in others, we speculate that the local inflammatory response contributes to the appearance of symptoms.

This study has a number of strengths. For example, fungal cultures were used to confirm the presence of current VVC and to classify the study participants into four distinct groups (RVVC, CNR, AS, and CTRL). We also collected important clinical information (including clearly defined symptom scores and clinical scores) for each study participant from whom vaginal tissue samples were obtained to evaluate correlations between clinical data and gene expression profiles. This study also has some limitations. The temporal dynamics of changes in gene expression between active episodes could not be proven, as this was a cross‐sectional study. Moreover, the AS and CNR groups were considerably smaller than the RVVC and control groups. Further research is warranted to explore factors associated with susceptibility to RVVC, as well as differences between VVC and AS colonization. Another limitation was the exclusion of women with bacterial vaginosis. As bacterial vaginosis has been linked to both an increased risk of acquiring RVVC and an increased rate of AS vaginal colonization with *C. albicans* [67, 68, 69], it would be interesting to examine the role of this condition as a confounding factor in the future. Hormonal factors, including regular menstruations and choice of hormonal contraceptive, also differed significantly between some of the study groups. This can potentially influence gene expression patterns, however, the small sample size of the AS and CNR groups as well as the fact that this data was based on self‐report through questionnaires which is less reliable than hormone measurements make it hard to draw any conclusions.

Overall, our results provide important insights into the transcriptional effects of RVVC on the vaginal mucosa, as well as the relationship between clinical severity and molecular inflammation in this condition. We found that the clinical severity of RVVC correlated with upregulation of genes involved in molecular inflammation and neutrophil activation. Furthermore, the vaginal mucosal transcriptome of women with RVVC who were between VVC episodes was similar to that of healthy controls, indicating that the molecular findings observed in women with RVVC are only present during active infection.

## Ethics Statement

The study protocol was approved by The Swedish Ethical Review Authority (2022‐03126‐01). Each participant provided written informed consent prior to any study‐related procedures.

## Conflicts of Interest

The authors declare no conflicts of interest.

## Supporting information



Supporting Information

Supporting Information

Supporting Information

Supporting Information

Supporting Information

Supporting Information

Supporting Information

## Data Availability

The raw sequencing data and clinical characteristics of study participants cannot be held in a public repository because of the sensitive nature of the data. Requests for data access can be made to the Karolinska Institutet Research Data Office (contact via rdo@ki.se), and access will be granted if the request meets the requirements of the institute's data policy. The processed sequencing data files can be accessed in the Gene Expression Omnibus public repository, accession ID GSE278036.
