# Supplementary material for: Vaginal Transcriptional Signatures of the Neutrophil‐Driven Immune Response Correlate With Clinical Severity During Recurrent Vulvovaginal Candidiasis
Source: Am J Reprod Immunol. 2025 Jan 7;93(1):e70040. doi: 10.1111/aji.70040 (PMC11706224; doi:10.1111/aji.70040)
Supplement: Supplementary file 1 — Supporting Information [file AJI-93-e70040-s006.docx]

|  | **RVVC**  **n=19** | **CTRL**  **n=18** | **AS**  **n=7** | **CNR**  **n=8** |
| --- | --- | --- | --- | --- |
|  | median or number  (range or %) | | | |
| Symptom score (0−5)^†^ | 4 (2−5) | 0 (0−1) | 0 (0−0) | 1 (0−3) |
| Clinical score (0−5)^‡^ | 3 (0−5) | 0 (0−0) | 0 (0−1) | 0 (0−2) |
| **Wet smears^2^** |  |  |  |  |
| Hyphae | 12 (63) | 0 (0) | 2 (29) | 0 (0) |
| Leukocytosis | 9 (47) | 1 (6) | 1 (14) | 0 (0) |
| Clue cells | 0 (0) | 1 (6) | 0 (0) | 0 (0) |
| Decreased amount of lactobacilli | 6 (32) | 5 (28) | 0 (0) | 1 (13) |

**Supplementary Table 1 Clinical characteristics of the study participants.** Women with

positive fungal cultures and ≥3 episodes of VVC per year were defined as an RVVC group, while women with negative fungal cultures and <3 episodes of VVC per years were defined as controls. Women with positive fungal cultures, symptom score = 0, and <3 episodes of VVC per year were defined as an asymptomatic group. Women with negative fungal cultures and ≥3 episodes of VVC per year were defined as a culture negative RVVC group. Symptom score: vulvovaginal discharge = 1p, itching = 1p, dryness = 1p, burn = 1p, pain = 1p, self-reported. Clinical score: vulvovaginal redness = 1p, discharge = 1p, dryness = 1p, fissures = 1p, hyphae on wet smear = 1p, as assessed by the examining gynecologist. Wet smears: Two wet mounts of vaginal discharge were stained with KOH and saline, respectively, and assessed by the examining gynecologist. ^†^Self-reported through questionnaires. ^‡^Assessed by examining gynecologist. Abbreviations: RVVC = recurrent vulvovaginal candidiasis, CTRL = controls, AS = asymptomatic, CNR = Culture negative RVVC, n/a = not available, na = not applicable, VVC = vulvovaginal candidiasis, KOH = potassium hydroxide.
