# Supplementary material for: Vaginal Transcriptional Signatures of the Neutrophil‐Driven Immune Response Correlate With Clinical Severity During Recurrent Vulvovaginal Candidiasis
Source: Am J Reprod Immunol. 2025 Jan 7;93(1):e70040. doi: 10.1111/aji.70040 (PMC11706224; doi:10.1111/aji.70040)
Supplement: Supplementary file 4 — Supporting Information [file AJI-93-e70040-s001.docx]

| **Cluster** | **RVVC**  **cluster** | **RVVC in diverse**  **cluster** | **All in diverse**  **cluster** | ***p*-value^†^** | | **Data n/a** | | |
| --- | --- | --- | --- | --- | --- | --- | --- | --- |
|  | median or number  (range or %) | | | RVVC/RD RD/AD | | RVVC RD AD | | |
| Age (years) | 30 (21−46) | 32 (24−42) | 30.5  (20−45) | 0.92 | 0.80 |  |  | 1 |
| BMI | 22  (21−27) | 21 (19−25) | 22  (16−36) | 0.37 | 0.74 |  | 1 | 4 |
| Smoker | 0 (0) | 0 (0) | 3 (7) | >0.99 | >0.99 |  |  | 2 |
| Healthy | 9 (100) | 10 (100) | 40 (95) | >0.99 | >0.999 |  |  | 1 |
| Atopy/allergies^‡^ | 7 (56) | 3 (30) | 13 (32) | 0.37 | 0.25 |  |  | 2 |
| Regular medications | 3 (33) | 3 (30) | 12 (29) | >0.99 | >0.99 |  |  | 2 |
| Cortisone drugs | 1 (11) | 1 (10) | 6 (14) | >0.99 | >0.99 |  |  | 1 |
| Dietary supplements | 5 (56) | 1 (10) | 10 (24) | 0.06 | 0.11 |  |  | 2 |
| Stable relationship | 5 (56) | 10 (100) | 33 (80) | **0.03*** | 0.19 |  |  | 2 |
| No. of pregnancies | 1 (0−5) | 2.5 (0−4) | 1 (0−5) | 0.97 | 0.57 | 2 |  | 5 |
| No. of children | 0 (0−3) | 1.5 0−3) | 0 (0−3) | 0.67 | 0.78 | 2 |  | 5 |
| Regular menstruations | 7 (78) | 6 (60) | 21 (51) | 0.62 | 0.27 |  |  | 2 |
| **Contraceptive use** |  |  |  |  |  |  |  |  |
| Hormonal IUD | 1 (11) | 5 (50) | 15 (36) | 0.14 | 0.24 |  |  | 1 |
| Birth controll pills | 5 (56) | 1 (10) | 10 (24) | 0.06 | 0.10 |  |  | 1 |
| No contraceptive | 3 (33) | 4 (40) | 16 (38) | >0.99 | >0.99 |  |  | 1 |
| **Previous gynecological infections** |  |  |  |  |  |  |  |  |
| Chlamydia | 4 (44) | 2 (20) | 10 (24) | 0.35 | 0.25 |  |  | 2 |
| Herpes | 1 (11) | 0 (0) | 1 (2) | 0.47 | 0.33 |  |  | 2 |
| Condyloma | 2 (22) | 2 (20) | 2 (5) | >0.99 | 0.14 |  |  | 2 |
| Foul-smelling vaginal discharge | 4 (44) | 1 (10) | 7 (17) | 0.14 | 0.09 |  |  | 2 |
| Cervical dysplasia | 3 (33) | 3 (30) | 9 (22) | >0.99 | 0.67 |  |  | 2 |
| Symptom score (0−5) ^¶^ | 4 (2−5) | 4 (2−5) | 0 (0−5) | 0.72 | <0.01 |  |  |  |
| Clinical score (0−5)^††^ | 4 (2−5) | 2 (0−4) | 0 (0−44 | 0.06 | <0.01 |  |  |  |
| Duration of RVVC  (years) ^¶^ | 2.5 (0.42−20) | 5.5  (3−17) | 5.5 (3−17) | 0.13 | 0.10 | 1 |  | 4 |
| **Fungal culture** |  |  |  |  |  |  |  |  |
| Azole resistance (%) ^‡‡^ | 0  (0−100) | 0  (0−50) | na | 0.08 | na |  |  |  |
| No. of azoles tested | 2 (1−5) | 2 (2−5) | na | 0.93 | na |  |  |  |
| **Wet smears^4^** |  |  |  |  |  |  |  |  |
| Hyphae | 8 (89) | 4 (40) | 6 (14) | 0.06 | <0.01 |  |  |  |
| Leukocytocis | 6 (67) | 3 (30) | 5 (12) | 0.18 | <0.01 |  |  |  |
| pH >4.5 | 1 (11) | 0 (0) | 0 (0) | 0.47 | 0.17 |  |  |  |
| Decreased lactobacilli | 4 (44) | 2 (20) | 8 (19) | 0.32 | 0.08 | 1 |  |  |

**Supplementary Table 4 Comparison of metadata between RVVC cluster and diverse cluster in UMAP.** Metadata from samples in the RVVC cluster was compared to metadata from RVVC samples in the diverse cluster and to metadata from all samples in the diverse cluster, respectively. Symptom score: vulvovaginal discharge = 1p, itching = 1p, dryness = 1p, burn = 1p, pain = 1p, self-reported. Clinical score: vulvovaginal redness = 1p, discharge = 1p, dryness = 1p, fissures = 1p, hyphae on wet smear = 1p, as assessed by the examining gynecologist. Wet smears: Two wet mounts of vaginal discharge were stained with KOH and saline, respectively, and assessed by the examining gynecologist. ^†^A Mann-Whitney U-test was applied for continous variables, and Fischer’s exact test was applied for binary variables. *P*<0.05 was considered statisticaly significant, significant *p*-values are marked with an asterisk. ^‡^Study participants reporting having either hay fever, eczema, asthma, food allergies, or drug allergies. ^¶^Self-reported through questionnaires. ^††^Assessed by examining gynecologist. ^‡‡^Azole resistance was assessed according to clinical routine, and measured as the prectentage of tested azoles the yeast proved resistant against. Abbreviations. RVVC = recurrent vulvovaginal candidiasis, RD = RVVC samples in diverse cluster, AD = all samples in diverse cluster, n/a = not available (excluded from statistical analysis), BMI = body mass index (kg/m^2^), IUD = intrauterine device, no. = number, KOH = potassium hydroxide.
