# Supplementary material for: Vaginal Transcriptional Signatures of the Neutrophil‐Driven Immune Response Correlate With Clinical Severity During Recurrent Vulvovaginal Candidiasis
Source: Am J Reprod Immunol. 2025 Jan 7;93(1):e70040. doi: 10.1111/aji.70040 (PMC11706224; doi:10.1111/aji.70040)
Supplement: Supplementary file 7 — Supporting Information [file AJI-93-e70040-s007.docx]

**Supplementary Table 2 Differentially expressed genes (FDR adjusted p-value <0.05) between study groups.** Abbreviations: RVVC = recurrent vulvovaginal candidiasis, CTRL = controls, AS = asymptomatic, CNR = culture negative RVVC, log2FC = log2(fold change), FDR = false discovery rate.

**Supplementary Table 3 Gene set enrichment analysis of all differentially expressed genes.** Abbreviations: GO = Gene Ontology, KEGG = Kyoto Encyclopedia of Genes and Genomes, MSigDB = The Molecular Signatures Database.

**Supplementary Table 5 Genes following the clinical score trend (FDR adjusted p-value <0.05).** Abbreviations: CS = clinical score, log2FC = log2(fold change), FDR = false discovery rate.

**Supplementary Table 6 Gene set enrichment analysis of the genes following the clinical score trend.** Abbreviations: GO = Gene Ontology, KEGG = Kyoto Encyclopedia of Genes and Genomes, MSigDB = The Molecular Signatures Database, CS = clinical score.
